# *Pectobacterium punjabense* Causing Blackleg and Soft Rot of Potato: The First Report in the Russian Federation

**DOI:** 10.3390/plants13152144

**Published:** 2024-08-02

**Authors:** Anna A. Vasilyeva, Peter V. Evseev, Alexandr N. Ignatov, Fevzi S.-U. Dzhalilov

**Affiliations:** 1 Department of Plant Protection, Russian State Agrarian University—Moscow Timiryazev Agricultural Academy, Timiryazevskaya Str. 49, 127434 Moscow, Russia; petevseev@gmail.com (P.V.E.); an.ignatov@gmail.com (A.N.I.); 2 Shemyakin-Ovchinnikov Institute of Bioorganic Chemistry, Russian Academy of Sciences, Miklukho-Maklaya Str. 16/10, 117997 Moscow, Russia; 3 Laboratory of Molecular Microbiology, Center for Precision Genome Editing and Genetic Technologies for Biomedicine, Pirogov Russian National Research Medical University, 117997 Moscow, Russia; 4 Agrobiotechnology Department, Agrarian and Technological Institute, RUDN University, Miklukho-Maklaya Str. 6, 117198 Moscow, Russia

**Keywords:** *Solanum tuberosum*, soft rot, blackleg, pectinolytic bacteria, *Pectobacterium punjabense*, genomic diversity

## Abstract

Phytopathogenic bacteria of the genus *Pectobacterium* are responsible for several diseases that affect potato (*Solanum tuberosum* L.) production worldwide, including blackleg and tuber soft rot. These bacteria are highly diverse, with over 17 different species currently identified. However, some of the recently described species, such as *Pectobacterium punjabense*, are still poorly understood. In this study, we focused on *P. punjabense* isolates collected from diseased potato tubers in Russia in 2021. Whole-genome sequencing was used to characterise the genomic diversity of the pathogen and determine the biochemical profiles of the isolated bacteria. The ability of these isolates to cause soft rot symptoms was tested. A comparative assessment of the potential pathogenicity of the *Pectobacterium* isolates was conducted by infecting potato tubers and measuring the accumulation of biomass in a liquid medium during cultivation at different temperatures. A TaqMan qPCR assay was developed for the highly sensitive and specific characterisation of *P. punjabense* strains, which can be used in diagnostic systems. This is the first report on *P. punjabense* causing potato disease in the Russian Federation.

## 1. Introduction

Potato (*Solanum tuberosum* L.) is a member of the *Solanaceae* family, which has the highest nutritional and commercial value among plants in this family [1]. At the same time, the main limiting factor to potato production is the proliferation of bacterial pathogens, which result in significant economic losses throughout the cultivation and post-harvest storage processes.

Potato blackleg and associated soft rot of tubers are among the most dangerous bacterial diseases caused by various species of pectolytic Gram-negative *Enterobacteriaceae* of the genera *Pectobacterium* and *Dickeya*. These pathogens have the ability to colonise an extremely wide range of host plants, including mosses [2] and numerous species of angiosperms plants, which contributes to their wide distribution range [3]. Pectobacteria is also capable of accumulating in the soil, the rhizosphere, and water bodies, persisting in epiphytic populations on the surface of cultivated and weedy plants, plant residues, and technological equipment. This contributes to the re-infection of plants growing in the field, as well as the infection of tubers from new harvests during harvesting, storage, and transport [4,5]. The pathogen penetrates plant tissues through natural or mechanical damage, mainly in high humidity conditions. The advent of genomic analysis and the pervasive utilisation of next-generation sequencing (NGS) to characterise bacterial strains have facilitated the identification of high genetic diversity [6] and the delineation of boundaries separating bacteria both between and within the genera *Dickeya* and *Pectobacterium* [7,8,9]. Nevertheless, some of the *Pectobacterium* species have undergone further taxonomic changes, resulting in the formation of new subspecies. For example, the *Pectobacterium carotovorum* species was previously highly heterogeneous, encompassing *Pectobacterium carotovorum* subsp. *carotovorum*, *Pectobacterium carotovorum* subsp. *odoriferum*, *Pectobacterium carotovorum* subsp. *brasilense*, and *Pectobacterium carotovorum* subsp. *actinidiae* [10]. To avoid inconsistency between the taxonomic status of species and subspecies within this large clade, subspecies have recently been elevated to species level [11], and new, previously unknown species have been described.

Thus, the genus *Pectobacterium* currently includes 17 described species: *Pectobacterium actinidiae* [11], *Pectobacterium aquaticum* [12], *Pectobacterium aroidearum* [13], *Pectobacterium atrosepticum* [7], *Pectobacterium betavasculorum* [7], *Pectobacterium brasiliense* [11], *Pectobacterium cacticida* [14], *Pectobacterium carotovorum* [11], *Pectobacterium fontis* [15], *Pectobacterium odoriferum* [11], *Pectobacterium parmentieri* [16], *Pectobacterium parvum* [17], *Pectobacterium polaris* [18], *Pectobacterium polonicum* [19], *Pectobacterium punjabense* [20], *Pectobacterium universal* [11], and *Pectobacterium wasabiae* [7], and two proposed species not yet confirmed by the ad hoc committee: *Pectobacterium peruviense* [21] and *Pectobacterium zantedeschiae* [22].

The timely detection and investigation of the distribution of new *Pectobacterium* species pathogenic to agricultural plants is of significant importance for disease monitoring, the improvement of molecular genetic methods for pathogen diagnosis, and the prevention of possible epiphytotics in potato cultivation regions.

Isolates of *Pectobacterium*, which were isolated from potato fields in the Punjab province of Pakistan in 2017, represent one of the recently described *Pectobacterium* species [20]. Some authors have classified this species as rare [23], yet it can be an important causing agent of blackleg and soft rot in potatoes. Furthermore, it is increasingly being found in different regions of the world. For example, a small percentage of *P. punjabense* strains were identified among other more prevalent pectolytic bacteria in symptomatic potato samples collected in Pennsylvania, USA, in 2018 [24]. Subsequently, the initial report of the detection of *P. punjabense* in China was published following an outbreak of potato blackleg in fields in Zhangjiakou, Hebei Province, in 2018 and in Ningde, Fujian Province, in 2019 [25]. Additionally, strains of *P. punjabense* were detected for the first time in Serbia in fields in Zobnatica in July 2019 [26] and in Ahome in the northwestern Mexican state of Sinaloa in January 2020 [27]. Furthermore, a phylogenetic analysis conducted recently revealed the presence of strains of *P. punjabense* in collections of phytopathogenic bacteria isolated in the USA between 2015 and 2016 [28] and in old European phytopathogen collections. For example, the Polish strain *P. punjabense* IFB5596 was isolated in 1996, indicating that this pathogen is not a fundamentally new species. Nevertheless, it remains a relatively uncommon occurrence, with the percentage of occurrence of this strain in European collections being less than 0.25% [23].

The present study was conducted with the objective of investigating the characteristics of *P. punjabense* strains that were first isolated in 2021 in the Russian Federation from potato tubers displaying symptoms of soft rot. This study included the characterisation of genomic diversity, produced on the basis of the whole-genome sequencing results. Biochemical tests were conducted, and the ability of the strains to induce blackleg symptoms in potatoes was confirmed upon inoculation on host plants. Furthermore, a comparative assessment of the pathogenicity of the isolates was conducted through the artificial infection of potato slices. The peculiarities of pathogen biomass accumulation during cultivation in a liquid nutrient medium at different temperatures were evaluated.

## 2. Results

### 2.1. Isolation and Phenotypic and Biochemical Characteristics of Bacterial Strains

In the course of work, various isolates of *Pectobacterium* sp. belonging to previously described species in the Russian Federation were isolated from potato tubers exhibiting symptoms of soft rot, and three samples of bacterial pathogens requiring further study were also isolated. The bacterial isolates were designated according to the potato varieties on which they were found. Thus, samples isolated from potato tubers of the Koroleva Anna variety (RS-3) were designated KA-4 and KA-5, while the isolate obtained from tubers of the Saturna variety (Elita) was named SATURN. Additionally, four strains of the most prevalent *Pectobacterium* species in Russia, sourced from the collection of the Laboratory of Molecular Bioengineering of the Shemyakin-Ovchinnikov Institute of Bioorganic Chemistry of the Russian Academy of Sciences, were utilised (Table 1).

Following a 24-h incubation period at 28 °C on a YD medium, the isolates exhibited shiny, rounded colonies of a cream colour. All three isolates exhibited morphological characteristics associated with *Pectobacterium* sp., demonstrated pectinolytic activity on CVP-SL medium and non-fluorescent properties when cultured on King B medium, and were also observed to be Gram-negative by Gram staining [29].

Biochemical features were employed as additional methods to differentiate the species of *Pectobacterium* (Table 2). All the strains under investigation were found to be positive for galactosidase and negative for urease, indole, and decarboxylase and did not produce hydrogen sulfide, which is generally consistent with the characteristics of *Pectobacterium* [30,31]. The strains were differentiated based on their activity towards tryptophan deaminase, gelatin hydrolysis, and their ability to form acid from melibiose. Concurrently, all strains demonstrated the capacity to oxidise glucose, sucrose, and amygdalin, as well as to reduce nitrate. Despite the general similarity in the biochemical profiles of different *Pectobacterium* species, *P. punjabense* strains exhibited unique biochemical characteristics. They differed from other species in their inability to oxidise mannitol and their ability to oxidise melibiose and demonstrated identical biochemical characteristics to each other.

### 2.2. 16S rDNA, Average Nucleotide Identity (ANI), and Phylogenetic Analysis

**Figure 1:**
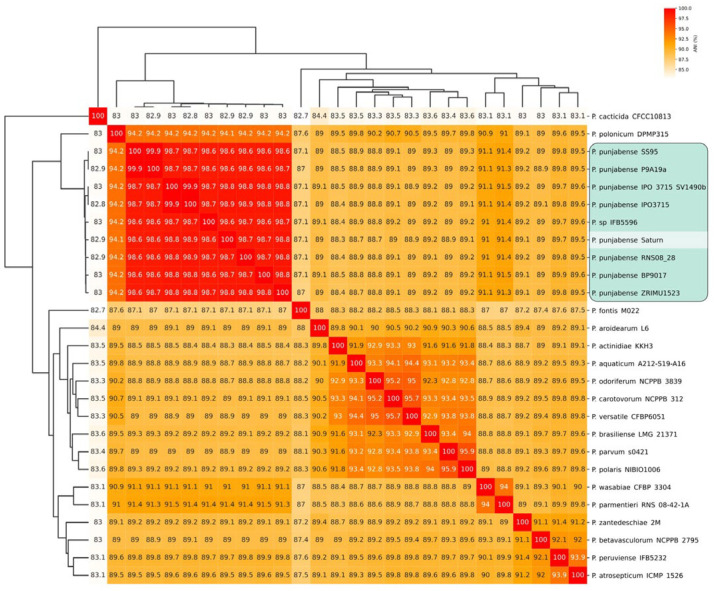
Similarity matrix and Neighbour-joining tree based on ANI values using genomic sequences assigned to the genus *Pectobacterium*. The matrix cells are coloured according to the ANI values on the scale in the upper right corner of the diagram. Strains that can be classified as *Pectobacterium. punjabense* are shown on a green background.

16S rDNA fragments were sequences that were compared to the closest sequences found with BLASTN. The nucleotide identity of the 16S rDNA of SATURN, KA-4, and KA-5 compared to *P. punjabense* SS95 (the type *P. punjabense* strain, NCBI GenBank accession number CP038498.1) was about 99.9%. The genome of strain SATURN was sequenced, and ANI calculations were performed using draft and complete genome sequences deposited in NCBI GenBank as of March 2024 and assigned to the genus *Pectobacterium*. Using the ANI values, the Neighbour-joining method grouped all known *P. punjabense* strains, *P.* sp. IFB5596, and strain SATURN in a distinct cluster, with ANI values of 98.6% and higher (Figure 1). Based on the prokaryotic species delineation threshold of 96% [32], strain SATURN (as well as *P.* sp. IFB5596) can be classified as a *P. punjabense* strain.

**Figure 2:**
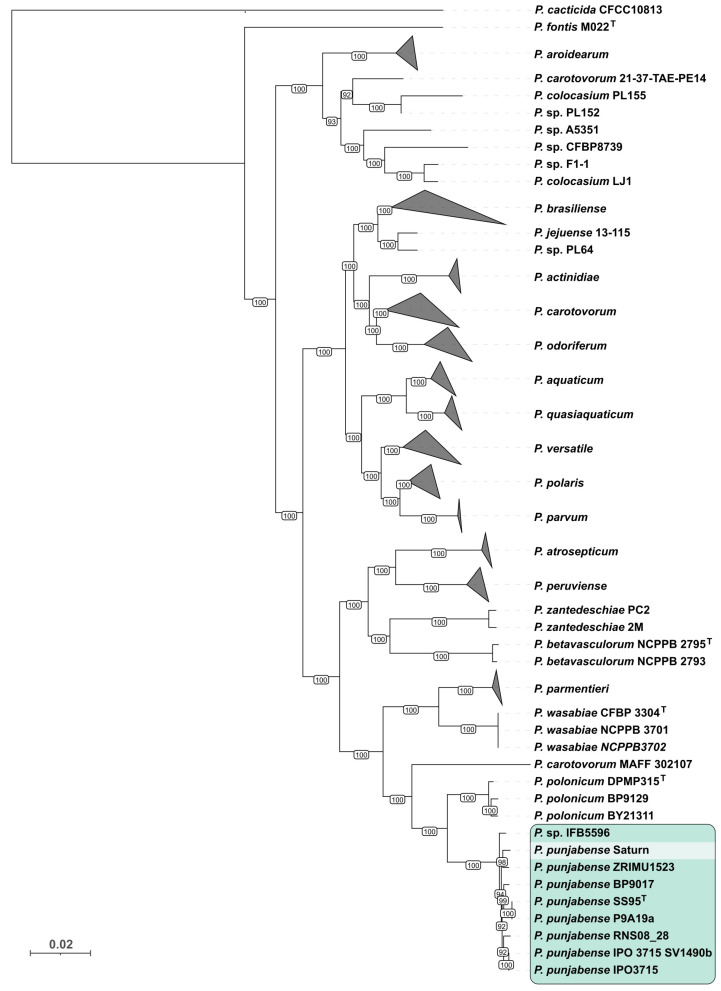
Maximum likelihood tree based on concatenated alignments of nucleotide sequences from 81 core genes using 465 *Pectobacterium* genomes and the genome of strain SATURN. Strains that can be classified as *P. punjabense* are shown on a green background. Branches containing more than 3 strains were collapsed; this tree with expanded branches is shown in Supplementary Figure S1. The numbers near the tree branches indicate the bootstrap support. The total number of bootstrap trees was 1000. The scale bar shows 0.02 estimated substitutions per site. The tree was rooted to *P. cacticida* CFCC10813. ^T^: Typical bacterial strains.

These taxonomic conclusions are supported by the results of a phylogenetic analysis based on concatenated alignments of 81 core gene sequences extracted from 465 *Pectobacterium* genomes downloaded from the NCBI Genome database (Figure 2) [33,34]. The tree places all *P. punjabense* strains, *Pectobacterium* sp. IFB5596, and strain SATURN in a distinct clade.

### 2.3. Development of a TaqMan qPCR Assay Specific to Pectobacterium punjabense

To develop a species-specific PCR diagnostic kit, a set of primers (Table 3) was constructed based on the gene sequence encoding *ShlB*/*FhaC*/*HecB* family of hemolysin secretion/activation-like proteins (NCBI GenBank accession number WP_010681414.1). This gene is present in a single copy in all earlier known *P. punjabense* genomes, as well as in the genomes of SATURN and *Pectobacterium* sp. IFB5596 and is related to the Type 5 Secretion System [35]. The gene is located adjacent to genes encoding cellulose synthase complex outer membrane protein, cellulose synthase complex periplasmic endoglucanase, and other proteins involved in cellulose biosynthesis, which is related to biofilm formation [36]. Biofilm-forming ability can significantly contribute to the virulence of phytopathogenic bacteria [37].

**Figure 3:**
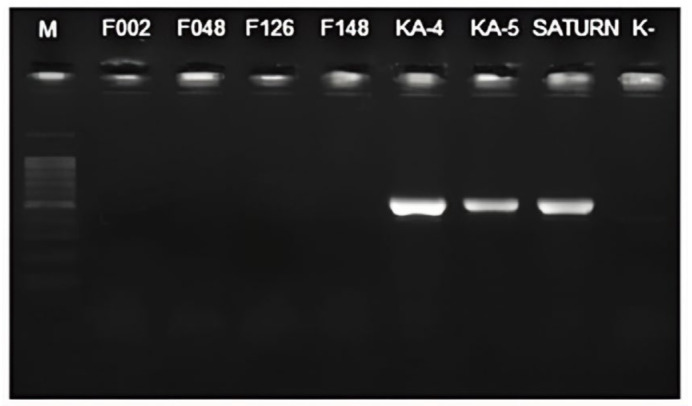
Specificity of the conventional PCR was visualised using a 1.5% agarose gel. The “1 kb DNA Ladder” (Evrogen, Moscow, Russia) was employed to ascertain the size of the amplicon.

The initial assessment of the species specificity of the designed primers yielded amplicons with the expected molecular mass of approximately 500 bp for the three target strains of *P. punjabense*. No nonspecific amplification products were observed (Figure 3). The sensitivity of the method was subsequently evaluated using TaqMan qPCR.

The specificity of the designed primers (PecpunR/PecpunF) and probe (Pecpun) was evaluated using a collection of previously isolated enterobacterial pathogens with blackleg and soft rot in the Russian Federation [38], which are phylogenetically related or potentially in the same ecological niche as *P. punjabense*. The TaqMan qPCR analysis yielded a strong signal (Ct values ranging from 27.56 to 28.17) with DNA extracted from *P. punjabense* strains but not with DNA from 12 other strains belonging to four different Pectobacterium species and three *Dickeya* species (all DNA calibrated at 10 ng/µL) (Table 4). Consequently, the Pecpun probe, in conjunction with the PecpunR/PecpunF primers, can be employed for the identification of *P. punjabense* bacteria.

**Figure 4:**
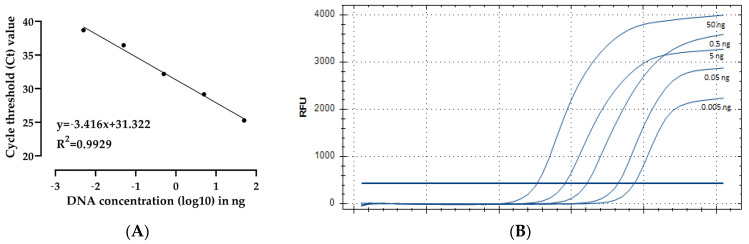
Sensitivity of *P. punjabense* DNA detection via real-time PCR: (**A**) standard curve showing the dependence of Ct on the concentration of pathogen DNA in the reaction, plotted against threshold cycles; (**B**) fluorescence curves obtained for a series of tenfold dilutions of genomic DNA from the SATURN strain.

To ascertain the sensitivity threshold, a series of tenfold serial dilutions of *P. punjabense* SATURN DNA, ranging from 50 ng to 0.005 ng, was employed. Figure 4A illustrates the standard curve for five serial dilutions of DNA analysed using TaqMan qPCR. A high negative correlation (R^2^ = 0.99) was observed between Ct values and purified DNA (Figure 4B). The slope of the curve was −3.416, corresponding to a PCR reaction efficiency of 96.21%, calculated in the linear zone, according to MIQE guidelines [39]. The assay demonstrated high sensitivity, with a limit of detection of 0.005 ng/µL genomic DNA and a Ct value below 40 (Table 5).

### 2.4. Pathogenicity of Pectobacterium Isolates on Tubers and Stems 

**Figure 5:**
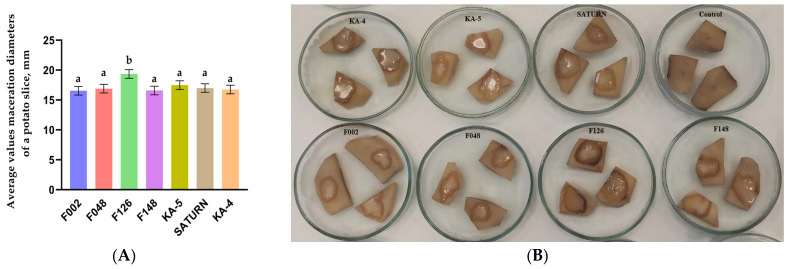
(**A**) Result of the average maceration diameters on potato (*Solanum tuberosum* L.) slices inoculated with potato blackleg and soft rot pathogens, mm. Different letters above the columns indicate groups with statistically significant differences (Duncan’s test, *p* = 0.05). (**B**) Symptoms of soft rot on potato slices observed 24 h after inoculation with *Pectobacterium* strains and incubation under anaerobic conditions at 28 °C.

Upon analysis of the pathogenicity of the strains on potato slices, all isolates exhibited pectolytic activity 24 h after inoculation. No maceration was observed on the negative control slices treated with sterile water (Figure 5B).

Statistical analysis of the averages revealed that the difference in damage caused by most of the strains was not significant. However, strain F126 of *P. brasiliense* exhibited the highest mean necrosis diameter of 19.3 mm (Figure 5A). For the remaining strains, maceration diameters ranged from 16.5 to 17.5 mm. 

Bacterial growth and multiplication can be considered an important pectobacterial virulence factor, which is highly dependent on temperature and population density [40]. The main objectives of this study were to evaluate the influence of three variables: temperature (16, 20, 24, and 28 °C), bacteria genotype (seven strains of four different species), and time after initial inoculation (24 and 48 h) on the maceration diameter of potato slices under controlled conditions. The results of the maceration dynamics calculation on inoculated potato slices indicate that, on average, for 48 h of incubation, the growth rate of the necrosis diameter was 0.81 mm/h. Concurrently, the greatest expansion of the maceration zone was observed during the initial 24 h of incubation at 28 °C. However, subsequent incubation of potato slices after 24 h till 48 h at this temperature was characterised by the lowest rate of necrosis increase (0.12 mm/h). The minimum rate of necrosis development over 48 h of incubation was recorded as expected at 16 °C, with a rate of 0.77 mm/h. Upon further examination of this incubation variant, it was found that during the incubation period of 24 to 48 h at this temperature, the maximum growth of necrosis was observed compared to other temperatures (0.26 mm/h). The reason for such temperature-specific dynamics can be in the development of the plant’s reaction against the pathogen or the accumulation of bacterial metabolites that suppress the pathogen’s growth. 

**Figure 6:**
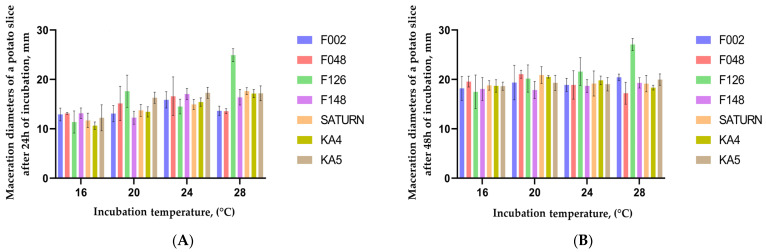
Diameters of maceration zones on potato slices inoculated with *Pectobacterium* strains at different incubation temperatures, mm: (**A**) results for slices incubated for 24 h; (**B**) results for slices incubated for 48 h. The values in the columns represent the mean values of three independent tests, and the error bars represent the standard deviations.

Figure 6A illustrates that the three *P. punjabense* strains, more than strains F002 (*P. versatile*) and F048 (*P. atrosepticum*) but less than strain F126 (*P. brasiliense*), showed increased aggressiveness under elevated temperature during 24 h after inoculation. Thus, the diameters of the macerated zone on potato slices inoculated with *P. punjabense* strains at 28 °C were from 25.7 to 29.4% greater than those inoculated with strains F002 and F048. However, measurements taken 24 h after inoculation at 16 °C showed a discrepancy of 5.7–23.5%, favouring the *P. versatile* and *P. atrosepticum* strains. 

The maximal maceration after 48 h was observed for strains KA-4 and SATURN at 20 °C, and for strain KA-5 at 28 °C (Figure 6B). With regard to strains F002 and F048, the maximum necrosis diameters observed over a period of 48 h of incubation were noted at 20 °C for the *P. atrosepticum* strain and at 28 °C for the *P. versatile* strain.

As it was described before, pectobacterial species exposed to their optimal temperature will have the advantage of maximum growth rate, allowing them to reach critical numbers first and activate enzyme production [41].

**Figure 7:**
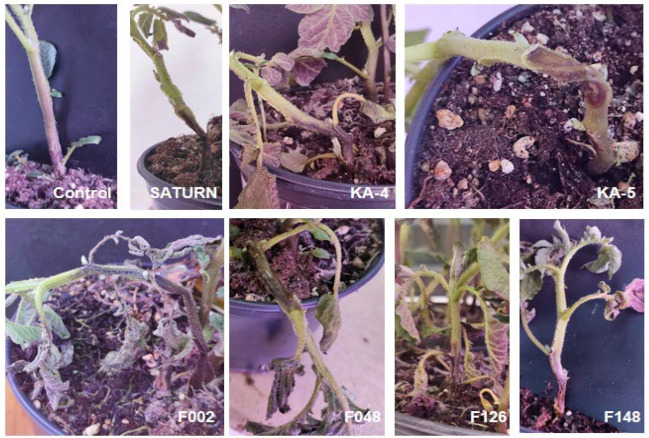
Blackleg symptoms observed in the stem tissue of potato plants 72 h after inoculation with *Pectobacterium* strains.

Upon inoculation of the host plants with the pathogen suspension, the typical symptoms of blackleg were observed, including blackening and water rot of the stem at the site of inoculation. Upon inoculation of the host plants with the pathogen suspension, typical symptoms of blackleg were observed, including blackening and water rot of the stem at the site of inoculation. These symptoms manifested 72 h after infection (Figure 7). The negative control plants, treated with sterile water, did not develop any disease symptoms. These symptoms manifested 72 h after infection (Figure 7). The negative control plants, which were treated with sterile water, did not develop any disease symptoms.

Bacterial strains reisolated from infected potato stem tissue exhibited biochemical and morphological characteristics consistent with those of *Pectobacterium*. The identity of the reisolated strains was confirmed via 16S sequencing, which is consistent with the postulates of Koch.

### 2.5. Cultivation in Liquid Nutrient Medium at Different Temperatures

**Figure 8:**
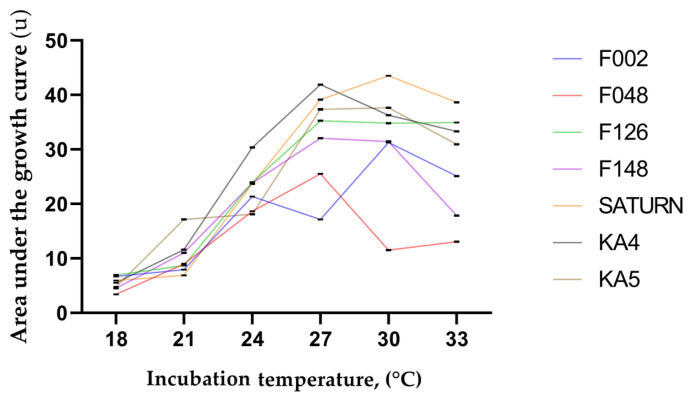
Graph of the area under the bacterial biomass growth curve, describing the population growth dynamics of *Pectobacterium* strains at different temperatures, according to changes in optical density in a liquid nutrient medium. The curves of the graph represent the mean values of three independent tests, and the error bars represent the standard deviation.

**Figure 9:**
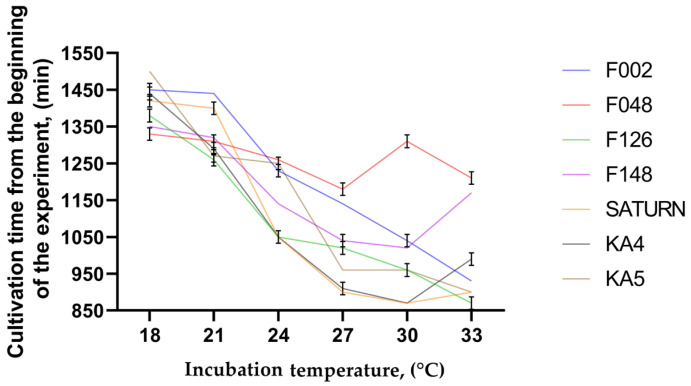
Graph of the curve reaching the exponential growth phase minutes from the beginning of the experiment, determined using the local maximum of the second derivative (the graph of the second derivative is not shown). The curves of the graph represent the mean values of three non-independent trials, and the error bars represent the standard deviation.

Following the cultivation of pathogens in a bioreactor with a liquid nutrient medium at different temperatures for 30 h, data on the change in the optical density of the medium were obtained, which were used to assess the growth of pathogen biomass. In control flasks, in which no bacterial suspensions were added before cultivation, the optical density remained unchanged throughout the experiments and was O.D._600_ = 0.0. The data obtained were employed to calculate the area under the biomass density growth curve (Figure 8) and the point of entrance into the exponential growth phase, which was determined using the local maximum of the second derivative of the bacterial density growth curve (Figure 9).

Figure 8 indicates that the SATURN strain exhibits the highest AUC value for optimal temperature among all strains at all analysed temperatures. This value, 43.5 u, was observed following cultivation at 30 °C. Two other *P. punjabense* strains also have AUC values exceeding the optimum characteristic for other strains, despite the fact that temperatures of 27–30 °C are optimal for all analysed strains. Thus, the peak AUC value for strain KA-4 is 41.9 at 27 °C, and for strain KA-5, it is 37.6 u at 30 °C. However, the exception is *P. brasiliense* F126, which shows a plateau effect starting at 27 °C. Consequently, when cultured at 33 °C, its optimum exceeds the AUC values for KA-4 and KA-5 by 4.86 and 12.9%, respectively. It is also noteworthy that *P. atrosepticum* strain F002 exhibits two optimum temperatures, at 24 and 30 °C. Additionally, the behaviour of *P. atrosepticum* strain F048 is worthy of note. This is the sole strain to demonstrate an increase in the area under the curve value of 13.36% from 30 to 33 °C.

A graph of pathogen biomass reaching the exponential growth phase (Figure 9) indicates that the maximum rate of infection growth is observed after 14.5 h from the beginning of the experiment when *P. brasiliense* strain F126 is cultured at 33 °C and *P. punjabense* strains SATURN and KA-4 at 30 °C. Among all incubation temperatures, strain KA-5 exhibited the fastest biomass gain at 33 °C, entering the exponential phase after 15 h from the beginning of cultivation. The minimum rate of entering the exponential growth phase within the range from 24 to 33 °C was observed when cultivating *P. atrosepticum* strain F048, with the minimum value observed at 30 °C after 1310 min from the beginning of the experiment.

## 3. Discussion

Blackleg is one of the most damaging and widespread bacterial diseases of potatoes, manifested by necrosis of the root part of the stems and soft rot of seeds or stored tubers. The causative agents of this disease belong to the *Enterobacteriaceae* family, which includes various species of pectolytic bacteria from the genera *Pectobacterium* and *Dickeya*. The high plasticity and ability to survive even under harsh conditions by parasitising many alternative hosts have prompted international concern about the economic losses caused by pectolytic bacteria to potato growers. In light of these concerns, there is a pressing need for further research aimed at monitoring and controlling the spread of these highly pathogenic microorganisms.

The advent of next-generation sequencing NGS technologies has facilitated a significant expansion of the understanding of bacterial genomics, including the genus *Pectobacterium*. It has become evident that this genus is highly diverse, and NGS analysis has facilitated the identification of strains in culture collections and allowed for the description of several new species of *Pectobacterium*. This is exemplified by the species *P. punjabense*, whose type strain SS95 was isolated from blackleg-affected potatoes in Pakistan in 2017 [42].

Currently, *P. punjabense* is arguably one of the least studied species of the genus *Pectobacterium*, which is pathogenic to potatoes. The number of studies on this species is relatively small, with a high degree of inconsistency in the findings. Cigna et al. [23] report that strain P9A19a, isolated in France in 2015, was found to be identical to the type strain SS95, isolated two years later in Pakistan. Concurrently, the oldest strain of *P. punjabense*, IFB5596, was isolated in Poland in 1996, which demonstrates the continued presence of this species beyond Pakistan for the past 25 years. Consequently, the true provenance of the species remains uncertain.

The rare occurrence of *P. punjabense*, combined with its pathogenicity (all strains of this species isolated so far from symptomatic potato plants), is certainly surprising. It is, however, important to note that the bacterial community present in rotting potato tissues is highly complex [43]. This may include several different pathogen species that act in a synergistic manner to suppress plant immunity but compete intensively for nutrients. The low number of isolated *P. punjabense* strains may be partly explained by their lower competitiveness [44], although this cannot be confirmed without differential identification of all *Pectobacterium* species. Moreover, numerous studies have demonstrated that the use of genus-specific primers Y1/Y2, traditionally employed for the identification of diverse *Pectobacterium* species, is ineffective in the case of *P. punjabense* strains [5,23,45]. This limitation impedes the prospect of detecting novel strains belonging to this species.

The present study examined three strains of *P. punjabense* isolated in 2021 from symptomatic potato tubers obtained from the Kemerovo and Moscow regions of the Russian Federation.

All isolates exhibited pectolytic activity during inoculation of host plants and potato slices at incubation temperatures ranging from 16 to 28 °C, thereby confirming their pathogenicity. Statistical analysis revealed that there were no significant differences in the aggressiveness of the studied strains.

The biochemical characteristics of the strains, as determined using API 20E panels (bioMerieux, Marcy I’Etoile, France), were found to be largely consistent with those previously described for *Pectobacterium* sp. [30,31]. However, all three *P. punjabense* strains exhibited a distinct inability to oxidise mannitol, which contrasts with the findings from certain studies on this species [26]. Conversely, in contrast to other *Pectobacterium* species, the *P. punjabense* strains did not exhibit the ability to oxidise melibiose. This is somewhat contradictory, as it is not consistent with the data describing the type strain *P. punjabense* SS95 [20], but is in agreement with studies by Cigna et al. [23]. In view of the above, biochemical tests cannot be used to differentiate *P. punjabense* strains from other *Pectobacterium* species. Nevertheless, biochemical tests remain a valuable tool for the characterisation and comparative evaluation of different strains within the *P. punjabense* species.

In order to ascertain the extent to which the strains under study were confined to specific temperatures, an experiment was conducted in which pathogens were cultivated in a liquid nutrient medium within a bioreactor at temperatures ranging from 18 to 33 °C. To determine the exponential phase of bacterial density growth, the second derivative was used to estimate the rate of infection growth within a certain time period, corresponding to the most intensive accumulation of pathogen biomass. This period was determined using two points of local extremal values of the second derivative, which correspond to the maximum concavity of the derivative and, consequently, the maximum convexity of the bacterial density growth curve [46]. This approach is the most adequate because it allows us to define the exponential phase within the limits of the highest growth rate rather than the entire growth graph, which is characterised by an intensive increase in infection at the beginning and a slowdown in growth towards the end of the exponential phase [47].

The results of the experiment demonstrated that the optimal temperature range for estimating the AUC across all strains was similar and fell between 27–30 °C. However, the maximum AUC values describing population growth dynamics were observed for the three *P. punjabense* strains, with the highest value observed for the SATURN strain. Concurrently, the rate of infection growth for the *P. punjabense* strains was greatest when incubated for 14.5 h at 30 °C for the SATURN and KA-4 strains and 15 h at 33° for the KA-5 strain. With regard to the *P. brasiliense* strain, this study indicates that it is the most closely related to the *P. punjabense* strains, both in terms of reaching the exponential growth phase at 33 °C and in terms of AUC values exceeding those of strains KA-4 and KA-5 when cultured at 33 °C. It can be proposed that *P. punjabense* may be more adapted to high temperatures than *P. versatile*, *P. atrosepticum*, and *P. parmentieri* but is either less or as active as *P. brasiliense*. This is a significant concern in light of recent climatic changes towards global warming, with an annual increase in mean annual temperature.

The preliminary clustering of the three strains into the *P. punjabense* group was based on the results of a comparative BLAST analysis of the 16S rRNA gene. Data obtained from genome assembly allowed the development of a TaqMan qPCR system with improved specificity and sensitivity for the *P. punjabense* species. For example, the previously developed qPCR system [23] reported a threshold Ct value of 34.52 at a DNA target concentration of 0.02 ng/mL in the reaction mixture. At a further reduction of the DNA concentration to 0.005 ng/mL, no positive results were obtained. In contrast, the TaqMan system developed in this study has a Ct value of 36.45 at a minimum DNA concentration of 0.005 ng/mL. Consequently, this assay can be employed as an efficacious instrument for the preliminary differential detection of *P. punjabense*, thereby facilitating the monitoring of the dissemination of this pathogen.

## 4. Materials and Methods

### 4.1. Bacterial Strains, Isolation, and Growth Conditions

This study employed three strains of *Pectobacterium punjabense* isolated in 2021 from potato tubers exhibiting symptoms of soft rot. This study also employed four other species of pectolytic bacteria (*Pectobacterium versatile* F002, *Pectobacterium atrosepticum* F004, *Pectobacterium brasiliense* F126, and *Pectobacterium parmentieri* F148) from the collection at the Laboratory of Molecular Bioengineering of the Shemyakin and Ovchinnikov Institute of Bioorganic Chemistry, Russian Academy of Sciences, as described earlier [38].

Isolation was performed from a pooled sample of 10 randomly selected symptomatic tubers. The tubers were surface disinfected by soaking for two minutes in a 75% ethanol solution, followed by three rinses with sterile distilled water. After that, cones were cut from the stolon part of the tubers without affecting the macerated areas and placed in a sterile 300 mL flask. The tuber pieces were then poured into 100 mL of distilled water and stirred on an ES-20 orbital shaker (Biosan, Riga, Latvia) at 150 rpm/min for 60 min at 28 °C. The homogenate obtained was seeded using serial tenfold dilutions on semi-selective pectate medium CVP-SL (the following components were used per 1 L of medium: CaCl_2_-2H_2_O—2.04 g; triptone—2 g; tris sodium citrate—2H_2_O—11.4 g; NaNO_3_—4 g; 0.1% crystal violet solution—3 mL; agar—8 g; NaOH (5M)—5.6 mL; pectin dipecta—36 g) [48]. Following a 48-h incubation period at 28 °C, bacterial colonies that cleaved pectin and formed characteristic pits on the surface of the nutrient medium were purified on YD medium (per 1 L of medium: yeast extract—10 g; dextrose—20 g; agar—17 g). The purified colonies were cultured for 24 h at 28 °C. Single colonies obtained were tested on King B medium (per 1 L of medium: yeast peptone—20 g; K_2_HPO_4_—1.5 g; MgSO_4_-7H_2_O—1.5 g; glycerol—10 mL; agar—15 g) to ensure the absence of fluorescence. The confirmation of Gram-negative status was achieved through Gram staining of the colonies. The strains were stored in a 15% glycerol solution at −80 °C until further studies could be conducted.

### 4.2. DNA Isolation

The isolation of DNA was conducted using a Phytosorb DNA extraction kit (Syntol, Moscow, Russia) in accordance with the manufacturer’s instructions, from an aqueous suspension of an overnight bacterial culture grown on King B medium. The quality and quantity of the extracted DNA were determined using a NanoDrop One spectrophotometer (Thermo Fisher Scientific, Waltham, MA, USA). The extracted DNA was stored at a temperature of −20 °C until further analysis.

### 4.3. Genetic Identification of Pectobacterium punjabense Strains

Classical PCR was conducted using a T100 thermal cycler (Bio-Rad, Hercules, CA, USA) in a 25 µL reaction volume. Each reaction consisted of 5 µL of 5× MasterMix (Dialat, Moscow, Russia), 0.2 µM of each primer, and 20 ng of matrix DNA.

Initial molecular identification was conducted using a set of primers for 16S rRNA gene amplification: 27F and 1492R [49]. The thermocycling regime consisted of an initial incubation at 95 °C for 3 min, followed by 29 cycles of denaturation at 95 °C for 30 s, annealing at 45 °C for 30 s, and extension at 72 °C for 45 s, with a chain extension at 72 °C for 6 min [49]. Amplicons, approximately 1200 bp in size, were visualised using electrophoresis in a 1.5% agarose gel supplemented with ethidium bromide in 0.5 × TBE buffer. A 1 kb molecular weight marker (Evrogen, Moscow, Russia) was used to determine the size of DNA fragments. The presence of a PCR product of the expected length was considered a positive signal.

The PCR fragments were purified from the reaction mixture using the ColGen kit (Syntol, Moscow, Russia) in accordance with the manufacturer’s instructions.

Sequencing of the purified PCR fragments was conducted using the BigDye Terminator v3.1 cyclic sequencing kit and the DNA Analyser 3730 automated sequencer (Thermo Fisher Scientific, Waltham, MA, USA).

### 4.4. Strain SATURN Genome Sequencing and Annotation

A starting material of 100 ng of genomic DNA, isolated using the Phytosorb DNA extraction kit (Syntol, Moscow, Russia), was used for the genome fragment library, as previously described.

The DNA was fragmented via ultrasound using a ME220 focused ultrasound machine (Covaris, Woburn, MA, USA) with the following parameters: iterations—7; duration—10 s; peak power—50; fill factor—20%; cycles per batch—1000.

Libraries were prepared using the Nextera DNA Flex library prep kit (Illumina Inc., San Diego, CA, USA) according to the manufacturer’s instructions. Sequencing was conducted on a MiSeq instrument using MiSeq V3 chemistry, achieving 2 × 300 nucleotide base pair (bp) reads. Reads were assembled using CLC Genomic workbench v. 7.5 (QIAGEN, Aarhus, Denmark) using default settings (coverage 108.0) and deposited in NCBI GenBank under accession number JBBWSM000000000. Binning was performed using MetaBAT v2 using default settings [50]. The assessment of completeness (98.66%) and contamination (0.55%) scores were carried out using CheckM v1.1.6 using default settings [51].

### 4.5. Calculations of ANI and Phylogenetic Analysis

Calculations of ANI, ANI clustering, and visualisation were conducted using fastANI v1.34 [51] and ANIclustermap (https://github.com/moshi4/ANIclustermap, accessed on 1 May 2024). Phylogenetic analysis was performed using UBCG2 [52] (under ‘nucleotide’ settings) and IQ-TREE v2.3.3 [53]. The IQ-TREE command line parameters were ‘--alrt 1000 -B 1000′ to provide an automatic determination of the best nucleotide substitution model and ultrafast bootstrap analysis using 1000 bootstrap replicates. Phylogenetic trees were visualised using iTOL v6 [54].

### 4.6. Development of PCR Diagnostic Kit

The diagnostic set of primers, including forward and reverse primers as well as the probe, was constructed with Primer3 using default settings [55].

#### 4.6.1. PCR Conditions

To initially assess the species specificity of the designed primers, conventional PCR was performed, with the subsequent detection of 518 bp amplicons in an agarose gel. The amplification parameters were as follows: primary denaturation at 95 °C for 15 min; then 35 cycles of denaturation at 95 °C for 90 s, annealing at 60 °C for 60 s, and elongation at 72 °C for 60 s; final elongation at 72 °C for 15 min. Each reaction was conducted in a volume of 25 µL and comprised 5 µL of 5× MasterMix (Dialat, Moscow, Russia), 0.5 µL of each primer (Table 3), and 20 ng of matrix DNA.

The PCR amplicons were separated using electrophoresis in a 1.5% agarose gel with ethidium bromide, and the results were documented using the GelDocXR+ imaging system (Bio-Rad, Hercules, CA, USA).

#### 4.6.2. qPCR

A TaqMan qPCR assay was developed for *P. punjabense* using a CFX96 Touch amplifier (Bio-Rad, Hercules, CA, USA). Thermocycling was performed with the following conditions: initial denaturation at 94 °C for 5 min, followed by 45 cycles of denaturation at 94 °C for 30 s, annealing at 60 °C for 10 s, and extension at 72 °C for 10 s. Each reaction contained 10 µL of 2.5× Mas ED Mix 0025 (Dialat, Moscow, Russia), 0.5 µL each of forward and reverse primers, 0.5 µL of the fluorescently labelled sample with 50-reporter dye [FAM-6-carboxyfluorescein (FAM)] and 30-terminally quenching dye (RTQ1), which were synthesised by Syntol (Moscow, Russia) (Table 3). The final concentration of each deoxynucleotide triphosphate (dNTP) was 0.2 mM, while the magnesium concentration was 1.25 mM. PCR water and 2 µL of matrix DNA were added up to a final volume of 25 µL.

The specificity of the designed primers was evaluated against three target strains and 12 previously identified related strains of different *Pectobacterium* and *Dickeya* species. The preparation of pure DNA samples was conducted in accordance with the methodology outlined in Section 4.2, with the samples subsequently diluted to a concentration of 10 ng/µL. A series of 10-fold serial dilutions of purified genomic DNA from the *P. punjabense* SATURN strain, spanning a range of 25 to 0.0025 ng/µL, were employed to assess the sensitivity of detection. Each dilution was employed as a matrix for real-time PCR.

PCR efficiency was calculated using the standard curve method, employing CFX Maestro™ 1.0 Version 4.0.2325.0418 software (Bio-Rad, Hercules, CA, USA). All experiments were performed in triplicate, in one run, and repeated three times, thus yielding nine technical repeats. As a negative control, reactions were performed with water without DNA addition.

### 4.7. Biochemical Characterisation of Pectobacterium Strains

The biochemical characteristics of each isolate were determined using a commercial API 20E assay system (bioMerieux, Marcy I’Etoile, France). The inoculum for the assay was obtained by selecting and suspending a single colony from a 24-h culture grown on YD medium in a buffer solution as part of a kit. The test system strips were incubated for 24 h at 28 °C following inoculation with the bacterial culture suspension. The test system included the following tests: ONPG (β-galactosidase production), ADH (arginine dihydrolase), LDC (lysine decarboxylase), ODC (ornithine decarboxylase), CIT (citrate utilisation), H2S (hydrogen sulphide formation), URE (urease production), TDA (tryptophane deaminase activity), IND (indole production), VP (acetoin production), GEL (liquefaction of gelatin), GLU (glucose fermentation), MAN (mannitol fermentation), INO (inositol fermentation), SOR (sorbitol fermentation), RHA (rhamnose fermentation), SAC (saccharose fermentation), MEL (melibiose fermentation), AMY (amygdalin fermentation), ARA (arabinose fermentation), and nitrite reduction to nitrate test. The experiment was conducted twice. The results were analysed and interpreted in accordance with the manufacturer’s instructions.

### 4.8. Pathogenicity Tests

Pathogenicity studies of *P. punjabense* isolates were carried out on potato tuber slices and stems of the host plants [27] of the Udacha variety in comparison with control strains *P. versatile* F002, *P. atrosepticum* F048, *P. brasiliense* F126, and *P. parmentieri* F148. A suspension of bacteria from an overnight culture grown on King B medium at 28 °C was used as inoculum. The optical density of the suspension was 0.2 at 600 nm, corresponding to 10^8^ CFU/mL, and was monitored using a NanoDrop One spectrophotometer (Thermo Fisher Scientific, Waltham, MA, USA) in OD_600_ analysis mode. The control was sterile distilled water.

In the potato slice assay, tubers were surface-sterilised with 75% ethanol, in accordance with the procedure previously described, and subsequently peeled and sectioned into 5 mm slices. The centre of each slice was damaged to a depth of 2 mm with a sterile toothpick pre-moistened in a bacterial suspension of pathogens. Subsequently, 10 µL of the inoculum was applied to the surface of each slice and placed in glass Petri dishes on four layers of moistened sterile filter paper. Sealed with food film, the Petri dishes with slices were incubated in a thermostat at temperatures of 16, 20, 24, and 28 °C over the course of a 48-h period. The diameters of the maceration zone were measured with an electronic caliper at 24-h intervals. The experiments were conducted in quadruplicate for each variant.

To investigate the effect of strains’ pathogenicity on the host, potato plants were cultivated at 20 °C and 70–75% relative humidity in 0.5 L plastic pots with a peat-perlite substrate (Veltorf, Velikie Luki, Velikie). The plants were cultivated on a plant growth rack, the STELLAR-PHYTO LINE (ANO AVTech, Moscow, Russia), with LED illumination of 5000 lux, a lighting regime of 16/8 h (day/night), and daily watering with equal volumes of water. At four weeks of age, the stems of the potato plants were injured with a sterile syringe needle at a height of 5 cm above soil level. A volume of 20 µL of the bacterial inoculum was injected into the stem, and the inoculation site was subsequently wrapped with Parafilm M laboratory tape (Amcor, Zurich, Switzerland) in order to prevent drying of the inoculum droplets, as previously described [56]. Plant accounting was counted 72 h after inoculation. For each isolate, control strain, and negative control, three shoots per plant were inoculated in triplicate.

At the conclusion of the experiment, Koch’s postulates were validated by reisolating *P. punjabense* strains from infected stem tissue on a CVP-SL medium and scattered to single colonies, in accordance with the previously described isolation protocol. The identity of the isolated strains was confirmed by comparing their biochemical and morphological characteristics with those of the original strains used for infection, as well as by sequencing the 16S gene and comparing the nucleotide sequences.

### 4.9. Cultivation in a Liquid Nutrient Medium

The growth of bacteria at different temperatures was monitored using a Biosan RTS-8 bioreactor (Biosan, Riga, Latvia). Pathogens were cultured in 50-mL centrifuge tubes filled with 15 mL of King B liquid nutrient medium. Prior to the commencement of the culturing process, 15 µL of the bacterial inoculum (O.D._600_ = 0.2), suspended in King B liquid medium, was added to each tube. An equal volume of King B liquid medium was employed as a control. The tubes were placed in the bioreactor and cultured at 1250 rpm/min for 30 h at temperatures of 18, 21, 24, 27, 30, and 33 °C. Optical density measurements were taken every 30 min. Each variant of the experiment was conducted in triplicate.

### 4.10. Statistical Analyses

The data were analysed using analysis of variance (ANOVA) [57] with the software Statistica 12.0 (StatSoft, TIBCO, Palo Alto, CA, USA). Mean values were then compared using Duncan’s *p* = 0.05 criterion [58].

The area under the bacterial growth curve was applied to analyse the quantitative summary of pathogen population intensity over time. It was used to calculate the average disease intensity between each pair of adjacent time points, as it was described elsewhere [59]. The second derivative of a function gives information about its curvature or whether it is concave or convex. The local maximal second derivative may indicate the temporal onset (the time when the maximal growth rate in biomass density is reached) [46]. The method of second derivative calculation was adapted from the works of Lelekov et al. [47] and Sanchez et al. [46].

GraphPad Prism 9.2.0 was used for plotting graphs.

## 5. Conclusions

Due to the high genetic diversity of potato blackleg pathogens, it is possible to monitor the spread of these pathogens only through the timely identification and detection of new species within the genus *Pectobacterium*, with subsequent improvement of methods for identifying these pathogens based on current phylogenetic analysis data. The discovery of *P. punjabiensis* in the territory of the Russian Federation has expanded our understanding of the true extent of this pathogen’s spread. Therefore, the development of the efficient TaqMan qPCR system described in this paper is essential for monitoring the pathogen’s spread. The biochemical characteristics of the strains, as determined using the API 20E panel, were generally consistent with those of *Pectobacterium* spp., except for the inability of *P. punjabense* to oxidise mannitol and its ability to oxidise melibiose. These findings may be of scientific interest in characterising and comparing different strains of *P. punjabense.*

## Figures and Tables

**Table 1:** List of bacterial strains used in this study.

Strain	Year of Isolation	Origin	NCBI GenBank Accession Number
*P. punjabense* SATURN *	2021	Kemerovo region	this study
*P. punjabense* KA-4 *	2021	Moscow region	this study
*P. punjabense* KA-5 *	2021	Moscow region	this study
*P. versatile* F002	2012	Moscow region	NZ_PDVY00000000.1
*P. atrosepticum* F048	2012	Tver region	NZ_PDDK00000000.1
*P. brasiliense* F126	2012	Samara region	NZ_RRYQ01000010.1
*P. parmentieri* F148	2013	Moscow region	NZ_PDDJ01000001.1

* Draft genome was sequenced and assembled in this study.

**Table 2:** Results of the analysis of the API 20E method for a panel of *Pectobacterium* species.

Chemical Reaction	*P. punjabense* *SATURN*	*P. punjabense* *KA-4*	*P. punjabense * *KA-5*	*P. versatile * *F002*	*P. atrosepticum* *F048*	*P. brasiliense* *F126*	*P. parmentieri * *F148*
β-galactosidase activity	+	+	+	+	+	+	+
Arginine dihydrolase activity	−	−	−	−	−	−	−
Lysine decarboxylase activity	−	−	−	−	−	−	−
Ornithine decarboxylase activity	−	−	−	−	−	−	−
Citrate utilization	−	−	−	−	−	−	−
H_2_S production	−	−	−	−	−	−	−
Urease production	−	−	−	−	−	−	−
Tryptophane deaminase activity	−	−	−	−	+	−	+
Indole production	−	−	−	−	−	−	−
Acetoin production (VP)	+	+	+	+	+	+	+
Liquefaction of gelatine	+	−	−	+	−	+	−
Fermentation of glucose	+	+	+	+	+	+	+
Fermentation of mannitol	−	−	−	+	+	+	+
Fermentation of inositol	−	−	−	−	−	−	−
Fermentation of sorbitol	−	−	−	−	−	−	−
Fermentation of rhamnose	+	+	+	−	+	+	+
Fermentation of saccharose	+	+	+	+	+	+	+
Fermentation of melibiose	+	+	+	−	−	+	−
Fermentation of amygdalin	+	+	+	+	+	+	+
Fermentation of arabinose	+	+	+	+	−	+	+
NO_3_ reduction to NO_2_	+	+	+	+	+	+	+

**Table 3:** Primers for the amplification of the species-specific region in *Pectobacterium punjabense*.

Primer/Probe	Nucleotide Sequence (5′–3′ Direction)	Amplicon Sequence for the Type Strain
PecpunF	CAC AAC CTT AAC AAT ACC GGC G	CAC AAC CTT AAC AAT ACC GGC GGT CAC CGC ACC AAC CAC AAG AGA TGC CGT CTG CTT CCC CAT CCA AAA AGT TGT CTT TCA TGA TGC AGA GTC GCT CCC AGC GAA AGA TCG CAC GGC CAT TCA GCA ACG CTA CCA AAG CCG CTG CCT TGA TTT AGC CAC AAT CCA TAA CGC CGT GAG GGA AAC CAC CAA TGC CTA CCT CAA TCG TGG CTT TGT CAC CAG TCA GGC CTA TTT ACA GGA GCA AGA CCT CTC CGG CGG CAC GCT CAT CAT CAG CGT CAG CGA GGG AAA GAT AGA AGC TAT TCG CAT GGA AGG GGA AAC GCC ACT CGC AAT CAA GAT GGC CTT CCC TAG GCT GGA AGGAC ATA TTC TTA ATC TGC GCG ACA TCG AAC AAG GGA TGG AAC AGT TGA ATC GTC TGC CTT CGC AGC AGG TTG CCA TTG ATA TTC AAC CGG GAA AAC AAG CAG GGA GTT CGA TTG TTT ATC TCA AGC GCA CCA CGC AAG CCC GTC CTG TCA CCC TCT CTC TCA GCG
PecpunR	CGC TGA GAG AGA GGG TGA CA	
ProbePecpun	(FAM)-TCA TGA TGC AGA GTC GCT CC-(RTQ1)	

**Table 4:** Specificity of the qPCR TaqMan for the detection of *Pectobacterium punjabense* in DNA extracted from strains belonging to various *Pectobacterium* and *Dickeya* species.

Strain	Geographical Origin	NCBI GenBank Accession Number	Detection in TaqMan Assay (Ct Value)
*P. punjabense* SATURN	Kemerovo	this study	28.04
*P. punjabense* KA-4	Moscow	this study	28.17
*P. punjabense* KA-5	Moscow	this study	27.56
*P. versatile* F002	Moscow	NZ_PDVY00000000.1	ND
*P. versatile* F016	Ryazan	NZ_RRYR00000000.1	ND
*P. versatile* F135	Moscow	NZ_PDVX00000000.1	ND
*P. atrosepticum* F048	Tver	NZ_PDDK00000000.1	ND
*P. atrosepticum* F162	Scotland	NC_004547	ND
*P. atrosepticum* F163	Belarus	NZ_CP009125	ND
*P. brasiliense* F126	Samara	NZ_RRYQ01000010.1	ND
*P. brasiliense* F157	Moscow	NZ_PJDL00000000.1	ND
*P. parmentieri* F148	Moscow	NZ_PDDJ01000001.1	ND
*D. solani* DFil	Voronezh	NZ_PGOJ00000.1	ND
*D. chrysanthemi* DSM 4610 ^T^	USA	GCA_000406105.1	ND
*D. dadantii* DSM 18020 ^T^	Comoros	NZ_CP023467.1	ND

^T^: Typical bacterial strains. Ct value: cycle threshold value, defined as the number of cycles required for the fluorescence signal to exceed the detection threshold. ND: not detected.

**Table 5:** Sensitivity results observed with qPCR TaqMan using a standard curve of stain *P. punjabense* SATURN DNA.

DNA Concentration	Ct Mean	Standard Deviation
50 ng	25.27	0.17
5 ng	29.15	0.87
0.5 ng	32.18	0.08
0.05 ng	36.45	0.05
0.005 ng	38.7	0.07
Control	ND	ND

## Data Availability

The data presented in this study are available on request from the corresponding author. In addition, the data that support the findings of this study are openly available in GenBank.

## Supplementary Materials

The following supporting information can be downloaded at https://www.mdpi.com/article/10.3390/plants13152144/s1, Figure S1: Maximum likelihood tree based on concatenated alignments of nucleotide sequences of 81 core genes using 465 *Pectobacterium* genomes and genome of strain SATURN. Strains that can be classified as *P. punjabense* are shown on a green background. The numbers near the tree branches indicate the bootstrap support. The total number of bootstrap trees was 1000. The scale bar shows 0.05 estimated substitutions per site.
