# Supplementary figures and images for: Pectobacterium punjabense Causing Blackleg and Soft Rot of Potato: The First Report in the Russian Federation
Source: Plants (Basel). 2024 Aug 2;13(15):2144. doi: 10.3390/plants13152144 (PMC11313954; doi:10.3390/plants13152144)

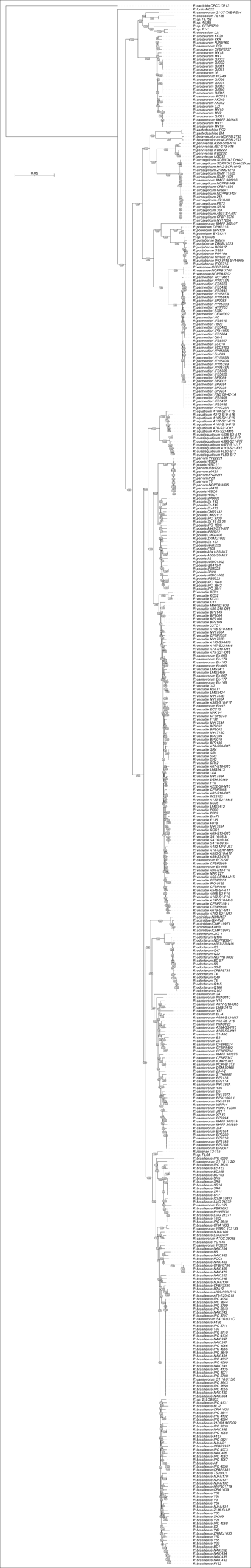

Supplement: Supplementary file 1 [file plants-13-02144-s001.zip › plants-3069632-supplementary.jpg]
